# Case Report: Integrating CBT, hypnosis-based consciousness activation techniques, and yoga-based postural training: a three-pillar approach used for migrant populations

**DOI:** 10.3389/fpsyt.2026.1737072

**Published:** 2026-06-26

**Authors:** Agnieszka Suchocka Capuano

**Affiliations:** Centre Hospitalier Sainte-Anne, Pôle Psychiatrie et Précarité, GHU Paris Psychiatrie & Neurosciences, Paris, France

**Keywords:** attention, CBT, consciousness, emotion, hypnosis, migrant, yoga, psychotherapeutic approach

## Abstract

Modern psychotherapy increasingly integrates multimodal and process-based approaches designed to respond to the complexity of contemporary clinical presentations. This paper introduces a three-pillar psychotherapeutic model combining (1) a revised cognitive behavioral foundation, (2) consciousness activation techniques derived from hypnosis, and (3) yoga-inspired postural and interoceptive training. This integrative framework was specifically adapted for migrant populations, who commonly present with emotional dysregulation, somatic complaints, dissociative symptoms, and limited access to long-term mental healthcare. These clinical challenges are often compounded by trauma exposure, linguistic barriers, precarious living conditions, and chronic stress. The model emphasizes present-moment emotional identification as a fundamental therapeutic process. Emotions are addressed not as targets for regulation or cognitive restructuring, but as perceptual signals enabling flexible behavioral adaptation. Intervention begins with the recognition of bodily emotional sensations, progresses through attentional detachment from non-current cognitions, and incorporates movement-based techniques to strengthen interoceptive awareness. In this framework, basic emotions—joy, sadness, anger, fear, surprise, and disgust—are conceptualized as biologically grounded and culturally universal, providing an accessible entry point for emotional work with culturally diverse patients. This paper proposes two intervention formats: (1) a transdiagnostic five-session psychotherapy focusing on emotional identification, interoceptive exposure, attentional training, and yoga-based postural work; and (2) a single-session “One-Shot Concentration Group” integrating mindful breathing with detached awareness and simple body–mind postures to enhance attentional flexibility. Both interventions were delivered with interpreter support when necessary and structured to minimize reliance on verbal processing, making them well suited for allophone patients. Preliminary clinical observations show immediate benefits such as increased attentional flexibility, reduced physiological tension, and enhanced mental calmness. Longer-term effects reported by patients and clinicians include improvements in sleep, concentration, memory, and decreases in anxiety, depressive symptoms, and dissociative episodes. These findings support the feasibility and acceptability of this three-pillar model in settings involving migrant patients facing multiple vulnerabilities. This work underscores the relevance of integrating cognitive, interoceptive, and movement-based psychotherapeutic elements, and highlights future directions for empirical evaluation, including the incorporation of objective physiological measures such as electrodermal activity to assess autonomic regulation and therapeutic impact.

## Introduction

Our work begins with a fundamental question regarding emotion regulation: What is it, and is it necessary to regulate our emotions? Gratz and Roemer (1) propose an integrative conceptualization of emotion regulation that extends beyond the modulation of emotional arousal. According to their model, emotion regulation also involves the awareness, understanding, and acceptance of emotions; the capacity to maintain control over one’s emotional responses and to engage in goal-directed behaviors even in the presence of so-called “negative” emotions; and the flexible use of emotion-regulation strategies adapted to contextual demands.

Our Three Pillar psychotherapy approach is the response for emotional regulation, which is treated as the capacity to cope (2–4) with everyday life issues from the following: (1^st^ Pillar) Beck cognitive therapy model (5), which analyzes situational cognitive and emotional links, detached mindfulness related to Wells (6), and activation of the Associative Emotional Network (7, 8 in 9). This flexible protocol recalls emotional, non-current memories, or, in the similar mode of Bower (10 in 11), related network dependent on emotions, cognition, and representations related to action. “An emotion possesses a verbal memory node, which is linked to other memory nodes associated with images, behaviors, expressive behaviors, or autonomic responses. The activation of an emotional memory nodes through verbal, perceptual, or imaginative experiences will activate associations between the different memory nodes (verbal, motor, autonomic). If a certain activation threshold is crossed, the memory content will become conscious”. (2^nd^ Pillar) Specific hypnosis-based consciousness activation techniques mobilize mental imagery involving the six senses, including proprioception. These techniques initiate the imaginary movement of the body in space (12). (3^rd^ Pillar) Yoga-specific training (13) introduces a perception of body in the movement and perception of attention, which guides this real actual movement here and now. The work of these three pillars is flexible and starts with body sensation identification through impersonal samples and progresses with hypnosis, yoga, and attention training toward bodily and mental flexibility.

Classical interventions from behavioral and cognitive therapies have their limits, particularly in relation to the feedback loop they activate, which is linked to emotional bodily sensations. According to Pierre Philippot (14), “Indeed, contrary to the postulate of the purist cognitive model, the issue here is not to criticize irrational thoughts at the propositional level, but rather to eliminate a feedback loop between automatic thoughts and a pathogenic schema”. This theory is also mentioned by Craske (15, 16).

Treating somatic symptoms and bodily sensations solely through interviews that engage the cognitive processing voice is not appropriate in emotional avoidance treatment (17, 18). This is especially true in the case of uncertainty life conditions like somatic disease recurrence or poverty, as in the population seen in our unit. Furthermore, the presence of ruminations and intrusive thoughts could reinforce behaviors of avoidance of emotional experience and consequently worsen the disorder. That is why new interventions are needed, first to cope with emotional avoidance, and second, to find a new way to communicate with non-French-speaking patients. Many cognitive behavioral therapy (CBT) interventions focus on a psychiatric disorder from the *DSM-V* (19) or, more recently, a group of disorders (transdiagnostic way) (20). Our concept focuses on psychological adaptation using Lazarus’ concept of coping (2–4, 21) and Ekman’s (22) model of basic emotion based on biological factors and related to facial expressions. These basic emotions are innate, intercultural, automatic, and short-lived. Symptomatic behaviors linked to a disorder can be different in each culture or each person; however, the basic emotion facial expression stays the same. That is why our interventions focus on emotion identification and exposure and not on post-traumatic stress disorder (PTSD), depression, or anxious disorders. The emotional function is independent of culture. This is an adaptive indicator to take consciousness of the present situation and find a freely chosen behavior.

In this publication, we propose an application of this approach of this three-pillar psychotherapy for migrant populations at risk of mental health problems. Patients from the migrant population present multiple psychopathological disorders with prevalences of 26.4% for depressive disorders (23) and anxiety disorders and 47% for PTSD (24), especially among refugees (25, 26). Recent studies have highlighted an accumulated prevalence of mental health problems in people living in disadvantaged social conditions compared to the general population (27–29) and in asylum seekers (30, 31). In particular, there are multiple symptomatologic manifestations of anxiety and depressive disorders listed in the *DSM-V* (19) often associated with PTSD. These are mostly sleep, attention, and concentration and memory disorders that impact adaptation to daily life. These symptoms are also present through somatoform disorders (19) such as pain or uncomfortable coenesthetic physical sensations (for example, anesthesia of a part of the body, heat, cold, stiffness, and tension). This therapeutic approach is based on a new theoretical model that operates transdiagnostically across complex patient presentations. Among migrant populations (32), the prevalence of somatization ranges from 12.9% to 67% (33).

### Context

The context of welcoming patients from the migrant population requires psychological interventions adapted to the needs of this specific population. CAPSYS is a psychiatric consultation service of the Precarity Unit of the GHU Paris Psychiatry & Neurosciences for migrants in precarious situations presenting with non-severe or chronic psychiatric disorders, such as anxiety, depression, or PTSD (34). It is a multidisciplinary team, notably with interpreter mediators in Dari, Farsi, Pashto, and Urdu. Face-to-face interpreters are used for the remaining languages. The population supported is made up mainly of young, allophone men, originally from Afghanistan but also from sub-Saharan Africa and Bangladesh. Recently (from 2024 and 2026), we observe more young women from Sri Lanka and Ukraine. They are mostly asylum seekers, living in the Ile de France region, in precarious conditions (50% on the streets) and often without resources. Psychotropic treatments, whose effectiveness is scientifically predictable and rapid, are the first-line therapeutic choice for anxiety-depressive disorders and PTSD, particularly for this population, often homeless and without resources, unable to attend regular consultations. However, in some patients, difficulties with concentration and attention are observed, as well as persistent ruminations and somatic symptoms, even after a clear improvement in mood, anxiety, or PTSD symptoms.

## Methodology

Our approach emphasizes present-moment emotional identification as a means of fostering autonomous and flexible behavioral choice. Emotion is conceptualized as a gateway to cognition rather than a target for modification. The focus remains on the experience of *current* thoughts, without attempting to alter their content. Through guided attentional disengagement—implemented without emotional avoidance—individuals learn to relate differently to their internal events and to adapt more effectively to situational demands. Emotion identification work allows to take consciousness of the presence of emotions, cognitive work helps to sort current thoughts from those that are not current, attentional training helps to detach attention from them without suppression, and yoga training helps to stay in the present moment through the attention that guides the movement.

We develop transdiagnostic interventions that integrate a shared core model with specific adaptations tailored to different patient populations (35, 36).

### Specific model and transdiagnostic framework

- Process-based therapeutic stance: Clinical work relies on continuous evaluation, careful attention to the unfolding therapeutic relationship, and empirical discovery within the session.
- Shift from traditional CBT: Rather than analyzing problematic situations or modifying dysfunctional cognitions, our process-based perspective focuses on identifying and working with underlying psychological processes, not symptoms.
- Targeting emotions Interventions address emotions arising in any situation—whether the patient initially identifies it as problematic—thereby engaging with emotionally salient contexts.
- Non-balanced view of emotions and cognitions: Emotions are no longer classified as positive or negative, and cognitions are not evaluated as functional, dysfunctional, or erroneous (cf. 5). Instead of searching for assumptions to correct, we cultivate attentional detachment from them while maintaining full emotional presence in the here and now and without connection with the emotional associative network (9, 37).
- We work on identifying emotions in the “emotional” situations of everyday life, not on “problem situations”.

**Figure 1:**
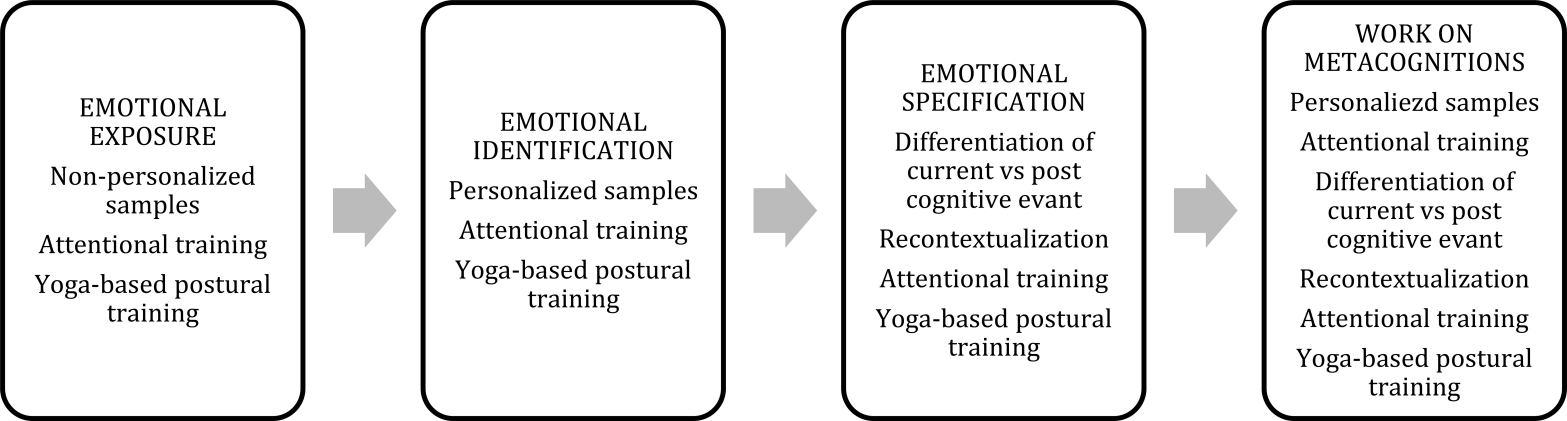
Diagram process of psychotherapy: a three-pillar approach.

The diagram process of psychotherapy: A-three pillar approach is described in **Figure 1**. Please note that yoga-training’s interventions are integrated from the second session of five sessions proposed (**Supplementary Appendix 3**).

### Groups

In response to the clinical needs identified within our patient population, two complementary psychotherapeutic interventions were developed: (1^st^) a single-session “One-Shot” program aimed at enhancing concentration and attentional and body flexibility, and (2^nd^) a five-session transdiagnostic program targeting emotional identification, emotional exposure, and interoceptive bodily awareness and flexibility.

(2^nd^) The “One-Shot” Single Session called “Concentration Group” focused on mindful breathing anchoring with detached awareness, attentional-training exercises (6), and six adapted yoga-based conscious-movement postures (**Supplementary Appendix 3**). The single-session intervention integrates visual and additive attentional-training procedures with yoga-inspired postures to address transdiagnostic attentional difficulties, and can be delivered with interpreter support (Dari, Pashto, French) under the supervision of a psychologist. Its objective is to strengthen attentional and concentration strategies and to facilitate the acquisition of adaptive coping skills, with sessions conducted either in a single language with translation or in multilingual settings depending on participants’ linguistic profiles. This unique group session allows for limited verbal interventions. The “One-Shot” intervention was designed for patients presenting anxious–depressive symptoms or with mild dissociative feature follow-up in hospital units with external consultations. This is an intervention proposed to treat minor psychiatric disorders’ symptoms and created for the purpose of mental health prevention for the group at risk of developing psycho-pathological disorders.

These transdiagnostic interventions manualized and progressively refined between 2013 and 2017 for both individual and group formats of 10 sessions were initially designed for patients presenting psychotic and bipolar symptoms and adapted for patients with anxious–depressive or mild dissociative feature symptoms, follow-up in hospital units, and external consultations. Their goal is to improve the ability to recognize interoceptive emotions and sensations and to integrate them with concomitant thoughts and mental images in order to support voluntary and adaptive behavioral responses (35, 36, 38).

(2) Transdiagnostic Psychotherapy: Emotional Identification, Exposure, and Interoceptive Sensations. The program used for migrant populations comprises five weekly sessions followed by a remote booster session at 1.5 months, delivered either individually (45 min) or in groups (90 min). This Group program called the “Emotion Group” at CAPSYS includes two structured modules—emotional exposure using images and sounds, and situational emotional identification—to develop adaptative coping in everyday life. Therapeutic support techniques include mindful breathing (39) anchoring with detached awareness, attentional training, and yoga training. The inclusion criteria are patients presenting with avoidance of emotional experience (denial and dissociation), showing little sign of improvement after taking medication, presenting with attention and concentration problems, and experiencing persistent sleep problems for more than 3 months.

### Assessments

As part of our current work, we offer patients the opportunity, at the end of each session, to participate in a brief discussion around a round table about their observations and perceptions regarding the group work or the individual session. Moreover, patients also discuss their group work feedback with psychiatrists or nurses during their follow-up appointments after sessions. In the current evolution of psychotherapy—particularly within this specific population—standardized questionnaires are not well adapted for assessment. In single-session or short-term interventions, we require evaluation tools that are easy to administer and minimally influenced by memory biases. We propose to conduct self-reported measures:

For individuals and groups, “Emotion identification group” interventions.

- The assessment of body flexibility (**Supplementary Appendix 4**), for example, by measuring how far the patient can extend their arms, quantified as the maximum distance they are able to reach when opening their arms, or move their arms: rolling their shoulders forward and backward (taking into account the physical problems observed by subsequent medical examinations).
- For filling a Beck (5) updated form (38) (**Supplementary Appendix 2**), the interval corresponding to the emotional situation and the completion of the form was noted. The loss of time between the situation and the act of filling in the form is correlated with an improvement in identification skills.
- Filling out the sleep diary. Fewer nighttime awakenings noted, and greater ease in falling back asleep after nighttime awakenings, demonstrates the effectiveness of the work accomplished.

For one-shot “Concentration groups”:

- We propose only the assessment of body flexibility, for example, by measuring how far the patient can extend their arms, quantified as the maximum distance they are able to reach when opening their arms, moving their arms, and rolling their shoulders forward and backward (considering the physical problems observed by subsequent medical examinations).

This individual assessment is proposed at the beginning and at the end of session.

### Population

A total of 204 individual psychological consultations were conducted between February and December 2025. Patients were referred by the consulting psychiatrist and presented with complex trauma accompanied by comorbid anxiety and depressive symptoms. Individual psychotherapeutic sessions were scheduled at intervals of 1 to 2 weeks.

For Group interventions from February 2025 to December 2025, 60 patients participated in “Concentration groups” and 22 patients participated in four “Emotion identification groups”, each composed of five sessions.

## Case descriptions

### Example of individual psycho-therapy appointments

#### Clinical case report—case no. 1: emotional processing and dissociative symptoms in a young adult migrant

##### Patient background

Ms. A is a 20-year-old woman from Sri Lanka, referred for a psychological evaluation by her psychiatrist due to difficulties with attention and concentration, as well as dissociative symptoms secondary to PTSD. These symptoms had been partially alleviated by psychotropic medication (paroxetine and alprazolam).

Ms. A arrived in France 6 months prior and had been followed at our Psychiatry Unit for the past 2 weeks. She is currently a university student in engineering and resides in a migrant shelter in the Paris region.

During the initial interview, the patient declined to discuss the circumstances that led her to flee her home country, referring only to her ongoing asylum application.

##### Session 1—initial clinical assessment

During the first session, Ms. A presented with a tense body posture, a sad facial expression, and a downward gaze. She reported sleep disturbances and concentration difficulties. Pronounced emotional avoidance and dissociative features were observed, including detachment from her body and her mental processes. Notably, the patient reported that she could not feel her arms or one of her legs.

A first intervention was proposed: a mindful breathing exercise using detached awareness (**Supplementary Appendix 1**). At the end of the session, the patient reported feeling calmer.

##### Session 2—emotional identification and somatic awareness

At the second session, Ms. A declined to discuss or draw her difficulties. We therefore initiated work based on identifying emotions through bodily sensations—locating the part of the body in which an emotion might be present. We then introduced a table illustrating the six basic emotions (38), consistent with Ekman’s (22) classification.

Although Ms. A remained reserved and spoke very little, she was able to identify one of her personal resources: dance. The session was adapted to her pace and focused on a personal learning process. A brief outline of proprioceptive support was provided without exploring the details of her emotional history.

To facilitate emotional engagement, we emphasized the presence of bodily sensations in a comfortable environment, drawing on consciousness activation techniques as described by Becchio and Suarez (12): *“to know them, to recognize them, and to use them when necessary”.*

At this moment, Ms. A mentioned her home country, Sri Lanka, and imagined herself surrounded by loved ones. When invited to perform a gesture if she wished, she spontaneously began moving her arms and performed a Tamil dance aligned with her emotional state.

##### Regulation techniques and clinical outcome

Following this moment of embodied expression, we introduced respiratory regulation techniques: pranayama-based slow-paced breathing (heart coherence breathing), followed by a dancer’s stretching routine.

The patient reported feeling calmer. We proposed continuing the heart coherence exercise to increase heart rate variability through slow-paced breathing (40).

Training in self-managed consciousness activation techniques is planned for the next session.

#### Clinical case report—case no. 2:

##### Patient background

Ms. B is a 28-year-old woman from India, working as a photographer and computer specialist. She reports taking family event photographs—particularly weddings—and describes having strong skills in photo editing and retouching. Since arriving in France, she has been living with her sister and her sister’s family in a small apartment with limited personal space.

Ms. B was referred by a psychiatrist following traumatic events that forced her to flee her home country. She has been prescribed paroxetine and alprazolam, which she reports taking regularly.

##### First interview

During the first encounter, Ms. B presented with limited motor engagement and frequent avoidance of eye contact. She described an event that occurred at home the day before the appointment.

We explored her emotional bodily sensations and asked her to associate these sensations with a color (a technique of reification to (41) render the sensation more concrete). Ms. B stated, *“It’s red”*, then added, *“It’s coming back”*, reporting fatigue, physical weakness, and rapid heartbeat—sensations she associated with anger. She expressed difficulty tolerating these bodily feelings, saying, *“Connecting to an emotional moment is difficult”.*

We explored her internal resources. Ms. B reported practicing yoga currently and since childhood. When asked to demonstrate a posture, she removed her shoes and spoke briefly about meditation. She performed a modified version of the Sun Salutation; her movements were very rapid, she trembled, nearly lost her balance, and said she had “lost the connection” with her body.

We invited her to sit cross-legged on the floor, facing the therapist, and to produce together the sound “om/aum” calmly and slowly (13). Afterward, Ms. B reported feeling calmer and soothed, and she smiled.

##### Second interview

During the second session, Ms. B reported experiencing nocturnal awakenings accompanied by sensations of cold and feeling her body “frozen”. She described one part of her body as feeling like “frozen meat”, with a dark blue color.

We explored her thoughts associated with this “cold and immobile body”. Ms. B described it as lifeless, without movement, and struggled to identify associated emotions.

Regarding her self-practice, she reported attempting yoga exercises but found it difficult to generate ideas on her own and said she felt fatigued. However, she expressed pleasure in attending the CAPSYS sessions.

We first proposed a simple yoga warm-up involving rubbing the hands together. When asked about the effect, Ms. B said: *“When we move, we warm up”*.

We explored her personal resources: previous tennis training, appreciation for nature, and a dislike of green.

Ms. B reported shoulder pain and connected it to a period during which she stated she had been captured. She described being forcibly seated and constrained.

We introduced the consciousness activation techniques exercise “Pain–Color” (12). She was able to delineate the painful area in both surface and depth. She described the color as dark blue gradually shifting to light blue and then to violet. When asked, *“What is violet?”* she responded: *“The sari is violet*”.

We proposed a proprioceptive-supported walk during which she moved while maintaining balance, imagining herself near a tennis court wearing a violet sari.

*Metaphor used*: “Green tennis balls bounce across the court while a young woman dressed in a violet sari walks forward toward the bright sun”.

We then introduced cardiac-coherence breathing (pranayama) and post-tennis athletic stretching.

Ms. B reported feeling soothed. We invited her to practice cardiac-coherence breathing autonomously at first. Learning of self-consciousness activation techniques is scheduled for the next session.

#### Clinical case report—emotional processing and somatic awareness (case no. 3)

##### Clinical context

Mr. C is a 19-year-old adolescent who arrived in France 1 year ago. He came alone and reports having no remaining family in his country of origin due to the death of his parents, severe violence in his community, and insufficient resources to live safely. He was referred for psychological support due to emotional dysregulation, somatic complaints, and difficulties identifying and managing internal experiences and dissociative symptoms (sensation to not to be present in his body, disconnection of present attention during social interaction interviews).

##### Session 1—initial assessment and introduction to mindfulness

During the first session, we explored the adaptive function of the six basic emotions, starting with a concrete emotional incident that had occurred the previous day. Mr. C expressed that: *“My emotion was very heavy very heavy like a strong weight”*.

We introduced a mindful breathing exercise based on detached awareness (**Supplementary Appendix 1**) (6, 39). At the end of the session, the patient reported a noticeable sense of calm.

##### Session 2—visual mindfulness and attentional shifting

During the second session, we proposed a visual mindfulness exercise inspired by Wells’ (6) detached mindfulness, practiced in natural conditions inside the consultation room. The patient was invited to observe the environment, including the view through the window, to shift attention and reduce cognitive fusion. The intervention aimed to facilitate attentional flexibility and distancing from intrusive thoughts.

##### Session 3—emotional and somatic identification

###### Presenting emotional and somatic symptoms

Mr. C described a lump in his throat and a heaviness in his shoulders. He associated these sensations with sadness. Using a visual chart illustrating the six basic emotions (modified Beck (5) Columns; **Appendices 2, 3**), we encouraged him to consider additional emotional states. While sadness was the first emotion he identified, he also pointed to fear, although he initially struggled to name it verbally.

###### Intervention: psychoeducation on basic emotions

We initiated a psychoeducational discussion to help the patient understand emotional bodily sensations as natural physiological experiences. The adaptive functions of the six basic emotions (9, 38) were reviewed:

- Joy: A transient and pleasant sensation triggered by positive or rewarding events, often shared socially. It increases heart rate, which limits its duration for physiological safety.
- Sadness: Provides a momentary pause that allows the body to rest and recuperate.
- Anger: Mobilizes energy to confront threat or initiate changes when a situation is perceived as unsatisfactory.
- Surprise: Creates physical and psychological distance, sometimes prompting a literal step backward to gain perspective.
- Fear: Supports flight or avoidance in the presence of perceived danger and helps individuals consciously navigate obstacles.
- Disgust: Protects the individual from consuming harmful substances or approaching situations that evoke instinctive rejection.

###### Clinical outcome of session 3

After linking bodily sensations to these basic emotional systems, Mr. C was able to clearly identify fear, an emotion he had previously indicated non-verbally but struggled to articulate.

##### Session 4—emotional identification and somatic regulation through yoga

During the fourth session, emotional identification work was continued with renewed focus on bodily sensations. We introduced yoga-based grounding and proprioceptive exercises to reinforce the perception of internal physical signals and promote emotional regulation. These interventions aimed to strengthen the patient’s interoceptive awareness and enhance his ability to differentiate emotional states through somatic feedback (**Supplementary Appendix 3**).

## Results

The first results stem from the observations of perceptions of patients after psychotherapy sessions in individual or group work. Patients reported the immediate effects like the sensations of attentional flexibility, mental appeasement, and feeling of lightness. In the delayed phase, patients reported improvements in sleep, memory, concentration, and learning. The health professional found improvements in sleep and reduction of anxiety and depressive symptoms, improvement of sleeping disorders, and reduction of occurrence of dissociative and delusion symptoms.

For example, Ms. A.’s assessments regarding the distance she can reach by opening her arms and by moving her arms (rolling her shoulders back and forth) before and after two psychotherapy sessions allowed her to observe that this distance was greater after the second session.

### Personal experience

From the CBT structured model.

I am very pleased to incorporate hypnotic work into my practice, as it complements cognitive–behavioral techniques for emotional identification and exposure, as well as yoga-inspired body–mind postural methods, which together constitute, in my view, a third pillar of modern psychotherapy. Before introducing any new technique to patients, I systematically practice it on myself. This allows me to develop greater familiarity and confidence with the method and enhances my motivation and therapeutic credibility after personally observing its effects.

Hypnosis provides access to techniques grounded in mental imagery, enabling therapeutic work through internally generated images that draw on the patient’s personal creativity. This approach, which integrates the imaginative capacities of both the clinician and the patient, supports dynamic, flexible, and continuously evolving therapeutic progress. The premise that “imagining an action can approximate performing it in reality” positions hypnotic imagery as a clinically relevant form of exposure or preparatory exposure, particularly useful in psychotherapy.

On a personal level, I have also observed the effectiveness of self-hypnosis and frequently discussed its benefits with colleagues, relatives, and patients. In yoga-training interventions, attention guides the movement and supports that movement in the chosen direction.

### Carried out interventions—what worked? And did not work?

Regarding the implemented interventions, we sought to determine which therapeutic components demonstrated effectiveness and which did not yield the expected outcomes.

### Temporality and context

According to the inclusion criteria described above, all interventions were successfully implemented. In cases of acute dissociative symptoms, the introduction of a psychiatric evaluation followed by an appropriate pharmacological treatment allowed patients to stabilize, become more comfortable, and engage more effectively in psychotherapy.

Some difficulties arose when patients were referred too rapidly to the psychologist. Our secondary hypothesis is that patients may not have sufficient time to develop initial spontaneous adaptive mechanisms after the first medical appointment. Moreover, each stage of the assessment process within a psychiatric unit for migrants is essential. The initial consultation—designed to evaluate both physical and psychological symptoms—helps patients develop awareness of their bodily and emotional experiences and recognize their need for psychological support.

### Personal motivation for change

As a third hypothesis, we considered the situation of patients presenting with a low level of motivation for change. Individuals with insufficient motivation, even when engaged in motivational interviewing, did not demonstrate clinical improvement comparable to that observed in patients receiving traditional CBT interventions. This suggests that low intrinsic motivation may limit the effectiveness of brief psychotherapeutic approaches, particularly in populations facing complex psychosocial stressors.

In this case, we propose to engage a specific work of motivation or leave a time for patients to think about an appropriate moment to introduce psychotherapy sessions. In this context, motivational interviewing drawing on decisional balance and structured interviewing techniques (42–44) is particularly valuable and useful. We also propose to gain experience through participation in a “One-Shot” Concentration group to enable adequate preparation for subsequent therapeutic interventions.

### What does not work?

There were not enough participants for emotion groups at the beginning of “Emotion groups”. We propose for the future to open sessions starting from 20 patients included in the group.

## Discussion

A systematic review and analysis of CBT brief psychological interventions for refugee and asylum-seeker populations (45) demonstrated a low immediate effect and low effect at 3 months post-intervention, as compared to a control condition. The authors noticed no difference between interventions conducted by mental health professionals and those by briefly trained peer refugees or other persons. These data can be interpreted as resulting from an overemphasis on CBT work and a lack of psycho-corporal behavioral, comportment-focused interventions.

That is why we need three pillars to support the psychotherapeutic work. First, from CBT: identification of body emotional sensations and labeling (15) without discussion or cognitive restructuring (38, 46). Second, hypnosis consciousness activation techniques and, third, yoga techniques adapted work with attention, which guided movement. These interventions were found to have a lot of positive effects on mental health status (47, 48).

In CBT, we typically work with patients to transform their expectations into specific, measurable, achievable, relevant, and time-bound (SMART) behavioral goals. These goals are set within a precise and flexible time frame, allowing for adjustments to the time frame in weeks or months (shortened or lengthened as needed).

In the evolution of modern psychotherapies, it is essential to emphasize the concept of adaptation and progress within the process, without necessarily expecting a positive outcome. The principle of “Expectation Violation” (15) leverages the feeling of surprise at unexpected success and actively challenges negative anticipations.

By considering a processual dimension, and particularly the progressive identification of emotions (with non-personal and then personalized content), we can distinguish between current and non-current emotions, to welcome current emotions within the present context, and to distance ourselves from cognitions related to parallel life events (35, 38). This ability to be in the present and to analyze the situation in real time allows us to choose our behavior freely and with full awareness. This act of sorting one’s own thoughts is possible thanks to the work of contextualization (placing oneself back in the present context by pointing to elements of reality) and the attentional detachment from parallel, non-current thoughts—seeing them like an airplane without seeking details about the type of aircraft, its composition, or its precise functioning. In this work, neither the emotional associative network (8 in 9) nor the memory knot (10 in 11) linked to non-current emotional memories in the form of mental images (49–51) is activated. We see our parallel thoughts as a white airplane lying far away in the air with colors, an orange airline logo, or the colors blue, white, and red logo. The avoidance of the emotional experience 20) is not engaged; the emotional feeling remains current and instantaneous, and therefore more comfortable.

We also wanted to raise the issue of problem behavior, a key concept in CBT. In clinical practice, during the exploration phase, we refer to a problematic behavior for the individual. However, subsequently, after conceptualizing the clinical case, we propose working on emotional situations (38) rather than problem situations (5). This approach of defocusing attention from the “problem” allows us to perform attentional training (6) and to perceive the full range of emotional moments and resources from the outset.

In the phenomenon of consciousness activation techniques linked to hypnotic processes, there is a “hyper-attentional” activation (52) of consciousness that complements and broadens the scope of work. We consider hypnosis, and more specially the current model of consciousness activation techniques, as one of the three pillars of modern psychotherapies. Following the activation of consciousness and attention, ideations that are otherwise difficult to access become more re-modulable during default mode functioning (53, 54), and thanks to the implicit therapeutic metaphors, deeper content becomes available for dynamic remodeling. Finally, this readjusted "guided discovery" (55) like in CBT clinical work (56, p.13-14) sets things in motion and provides the rhythm to "dance to the beat of emotions"! 12, p.28).

For centuries, humans have sought to control their environment and tame their emotions, often leading to maladaptive responses to themselves and their physical and social surroundings. Emotional regulation implies the concept of control. In the other way, we do not need to control anyway.

Regulating one’s emotions: is it necessary? If we can identify our emotion and accept occurrence of emotion manifestations, in particular the body’s sensations and link our emotion to our current thoughts, we can freely choose our actions. In this case, we do not need to regulate any emotion. We need to train our attention to detach it from our outdated cognitions and see them far away.

We do not work on problematic situations but on situations that mobilize emotion situations with comfortable or uncomfortable body sensations and emotions. By considering a processual dimension, and particularly the progressive identification of emotions (with non-personal and then personalized content), we can distinguish between current and non-current emotions, to welcome current emotions within the present context, and to distance ourselves from cognitions related to parallel life events (38, 46). This ability to be in the present and to analyze the situation in real time allows us to choose our behavior freely and with full awareness. This act of sorting one’s own thoughts is possible thanks to the work of contextualization (placing oneself back in the present context by pointing to elements of reality) and the attentional detachment from parallel, non-current thoughts—seeing them like an airplane without seeking details about the type of aircraft, its composition, or its precise functioning. In this work, neither the emotional associative network (8 in 9) nor the memory knot (10 in 11), linked to non-current emotional memories in the form of mental images (49–51), is activated. We see our parallel thoughts as a white airplane lying far away with colors, an orange logo, or the blue, white, and red colors. Avoidance of the emotional experience (20) is not engaged; the emotional feeling remains current and instantaneous, and therefore more comfortable.

Our interventions are in the same direction as Preece’s (57) propositions of the new perspective for the future work on emotion regulation, namely, (1^st^) emotional identification—increasing the number of studies; (2^nd^) increasing focus on the flexibility and adaptive function of emotions; (3^rd^) regulating “positive” emotions in the psychopathological context; (4^th^) emotional regulation across cultures (adaptation of occidental concept); (5^th^) development of new psychometric assessment tools; (6^th^) research and development of new theories on emotional adaptability or a return to older theories that can influence the emotional regulation process; (7^th^) alexithymia is a concept created by Sifneos in 1972, described by Bertagne et al. (58), considered a trait that could impair emotional regulation; and (8^th^) beliefs about self-efficacy in activating emotional regulation influence emotional regulation and adaptation.

This approach may be applicable to the management of complex clinical cases, particularly among patients with frequent PTSD diagnoses, and relies on relatively brief psychotherapeutic interventions. These interventions could be One-Shot, two to six sessions to provide a significant effect on everyday life adaptation.

Our proof of concept works because:

“In comparison to cognitive reappraisal of thoughts, distraction, and exposure alone, affect labeling during exposure was found to reduce skin conductance and increase approach behavior at one week follow-up in a context different than the exposure context” (Kircanski et al., in 15: p. 15).

### Progression of the proofs of efficacity of three-pillar therapeutic interventions

Subjective assessments of these symptoms and their intensity are difficult to carry out given their link to the sympathetic nervous system, which functions automatically and is therefore mostly non-conscious. According to Boucsein (2012 in 59), “as individuals are not always aware of these stimuli, phasic signals would make it possible to measure unconscious tension and arousal” (60, 61).

Moreover, for efficiency evaluation, these new interventions need a modern data collection method different from questionnaire using limits. According to the literature review proposed by Ikawa (62), “One of the indicators of the state of the autonomic nervous system is electrodermal activity (EDA). EDA represents variations in the electrical conductivity or resistivity of the skin surface, fluctuating in response to the activity of eccrine sweat glands, controlled by the sympathetic nervous system (63). Sweating variations are triggered by ambient temperatures and central nervous system activity, linked to emotional and cognitive states (64, 65). EDA therefore has potential as a simple biomarker of depression, as it reflects the activity of the sympathetic nervous system and allows for noninvasive measurement”.

Additionally, Kim (59) and colleagues highlighted that “(…) electrodermal activity (EDA), which reflects the activity of the sympathetic nervous system, is also sensitive to changes in clinical status (66). Specifically, depressed patients had lower resting skin conductance levels (SCL) than healthy control subjects (67). Similarly, stress-induced autonomic arousal, as measured by EDA, was significantly reduced in patients with Major Depression Disorder (MDD), indicating that depression may be associated with decreased autonomic responses to stimuli (68, 69). Moreover, EDA in participants with MDD was distinguished from that of patients with other psychopathologies, such as generalized anxiety disorder (GAD) or panic disorder (PD), as individuals with both GAD and PD tended to exhibit autonomic hyperactivation (68, 69)”. It is therefore necessary to conduct the modern and tailor-made interventions considering recent objective data.

### Perspectives of development of interventions

We propose conducting more targeted assessments to guide patients toward interventions that best match their needs. For example, patients with a low level of psychological symptoms of anxiety and depression or somatoform symptoms could be directed to mental health preventive interventions preferably with a group. This prevention-focused approach aimed at reducing the onset, persistence, or worsening of PTSD symptoms requires fewer psychotherapeutic or medical interventions and is less costly in terms of time and resources. In contrast, patients with complex or multiple traumas should be referred to individual psychotherapy.

In the future, we plan to continue administering individual assessments and to implement additional evaluations based on objective biological measures. Electrodermal activity (EDA) will be recorded using devices such as Embrace2 for Research (62) or the EDOR system (59, 70).

EDA is a well-established indicator of autonomic nervous system activity (62). EDA reflects variations in the electrical conductivity or resistivity of the skin surface, which fluctuate in response to eccrine sweat gland activity under sympathetic nervous system control (63). These fluctuations are influenced by ambient temperature as well as central nervous system processes associated with emotional and cognitive states (64, 65). Given these properties, EDA constitutes a promising noninvasive biomarker of stress and mental distress, as it directly reflects sympathetic activation.

## Conclusion

Three pillars of modern psychotherapy—cognitive and behavioral revisited background, consciousness activation techniques, and yoga-inspired training incorporating postural awareness work—are necessary to conduct the efficient intervention of psychotherapy especially in the migrant population. Our interventions are appropriate, based on CBT-, hypnosis-, and yoga-adapted interventions related to recent discoveries of neurosciences (71). They are necessary for the allophone migrant population and demonstrate a positive perception among patients and mental health professionals. We will soon conduct further subsequent studies to evaluate the effectiveness of our interventions using objective modern measures.

## Data Availability

The original contributions presented in the study are included in the article/**Supplementary Material**. Further inquiries can be directed to the corresponding author.
