# Supplementary material for: Case Report: Integrating CBT, hypnosis-based consciousness activation techniques, and yoga-based postural training: a three-pillar approach used for migrant populations
Source: Front Psychiatry. 2026 Jun 26;17:1737072. doi: 10.3389/fpsyt.2026.1737072 (PMC13350056; doi:10.3389/fpsyt.2026.1737072)
Supplement: Supplementary file 1 [file Supplementaryfile1.pdf]

“The guide to mindful breathing with integrated detached awareness”

Adapted by: Agnieszka Suchocka Capuano

"Please sit comfortably in your chair for a moment. I propose you keep your back straight, your shoulders relaxed and toned, and your chin tucked in as much as possible. You can imagine a magic thread holding your head and spine straight and comfortable. When doing this exercise at home, you can lie down. Now, gradually you can close your eyes in rhythm with your breathing, or you can fix your gaze on a point in front of you, or look down at the floor. You are free to choose whatever option feels most appropriate for you at this moment, and you may change your choice at any point during the exercise.

If you are sitting, your feet should be flat on the floor. Your hands should rest on your thighs, with your palms in a natural position.

Feel free to adjust your posture at any time, speak, or move if needed.

You can feel your feet firmly planted on the hard floor. You can feel the hard floor beneath your feet. You can feel the texture of your clothing through your hands resting on your legs. You can feel your body temperature through your clothes. You can touch your legs to feel it.

Your attention is focused on your breath. It will guide you throughout this exercise. You are here, in this moment, you breathe in and breathe out naturally.

Sometimes during this exercise, you may feel cool air entering your nostrils and slightly warmer air leaving them. Focus on your breath and let it flow naturally at its own pace.

During this exercise, you might notice sounds from nearby or outside. Let them come to you; remain in the present moment. Your attention is focused on your breath, which guides you throughout this practice. Your breathing is instinctive, spontaneous, natural. You feel the posture of your body sitting on the chair. The points of contact between your body and the seat.

You may also experience bodily sensations or emotions that arise within you. Simply observe them without trying to analyze or understand them further, without attempting to assign them any meaning. Allow them to manifest in your body, beginning with the top of your head, your skull, your face, your neck, your shoulders, your upper back, your ribcage, your arms, your lower back, your abdomen, your upper legs, the middle of your legs, and finally your lower legs, moving from your feet. These bodily sensations are instantaneous and spontaneous. They pass through your body and disappear. And there you are, sitting in the chair in the consultation room, on (date), breathing at your own pace. It is your breath that guides you throughout this process.

Sometimes, during this exercise, thoughts may arise. Observe them as they naturally appear, like white clouds drifting across a blue sky. Your attention is focused on your breath, which is natural and spontaneous. For a few moments, continue this breathing exercise on your own, at your own pace. Thoughts and images pass by, and you feel your feet flat on the ground, anchored to the

hard earth. You feel the hard ground beneath your feet. Observe thoughts or images that appear in your mind, and your attention can freely move from one thought to another or from one image to another. You can voluntarily engage and disengage your attention as you wish. It is you who decides to focus your attention on one object or another. Then the object of your choice will appear more clearly, and the other objects will appear more blurred. You see the entire landscape and all the thoughts that are part of that landscape, and you can then freely and intentionally choose to focus your attention on a specific thought. You are here, sitting in the present moment, breathing spontaneously. I now invite you to continue this exercise on your own for a few moments.

Now, gradually reconnect with your surroundings and gently introduce some movement into your body. When you feel ready, you can move your fingers, toes, hands, feet, and legs. You can move your head, turning it right and left, forward and backward, respecting your physical limitations. You can also move your arms forward and backward, and roll your shoulders, first one shoulder separately, then both shoulders together. You can yawn, sigh, and stretch as you wish. I now invite you to open your eyes at your own pace to become aware of your surroundings and conclude this exercise."
