# Supplementary material for: Case Report: Integrating CBT, hypnosis-based consciousness activation techniques, and yoga-based postural training: a three-pillar approach used for migrant populations
Source: Front Psychiatry. 2026 Jun 26;17:1737072. doi: 10.3389/fpsyt.2026.1737072 (PMC13350056; doi:10.3389/fpsyt.2026.1737072)
Supplement: Supplementary file 2 [file Supplementaryfile2.pdf]

<u>Instructions:</u> Fill in the columns after experiencing an emotional situation when you were alone or during a social situation that you observed or participated in. It is important to complete the columns no later than 30 minutes after the emotional situation.

**Situation**	**Emotions**	**Body's Sensations / interoceptive sensations**	**Feeling Related to several emotions**	**Thoughts**	**Mentals Images**	**Behaviors**
Date..<br>Hour...<br>Place..<br>Event (description)...............................<br>..<br>...	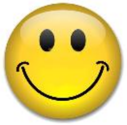<br>1. □ :<br>..................................<br>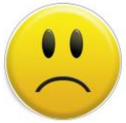<br>2. □ :<br>..................................<br>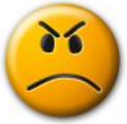<br>3. □ :<br>..................................<br>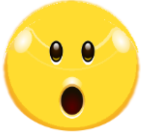<br>4. □ :<br>..................................<br>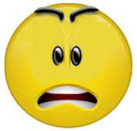<br>5. □ :<br>..................................<br>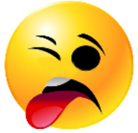<br>□ :<br>6................................					

Adapted by: Agnieszka Suchocka Capuano
