# Supplementary material for: Case Report: Integrating CBT, hypnosis-based consciousness activation techniques, and yoga-based postural training: a three-pillar approach used for migrant populations
Source: Front Psychiatry. 2026 Jun 26;17:1737072. doi: 10.3389/fpsyt.2026.1737072 (PMC13350056; doi:10.3389/fpsyt.2026.1737072)
Supplement: Supplementary file 3 [file Supplementaryfile3.pdf]

6 Psycho-corporal Postural Work Positions:

Elaborate by: Agnieszka Suchocka Capuano & Sylvie Rallon

Postural Body Work – Instructions

These exercises, inspired by yoga, aim to:

- Help you become aware of your body and your breathing in the present moment, here and now;

- Teach you to recognize your body, feel its limits and flexibility;

- Promote balance, concentration, and memory;

- Help you manage your emotions and bodily sensations;

- Allow you to learn certain exercises you can repeat in your room or at home.

Important points for this practice:

- No competition—neither with yourself nor with others;

- Be kind to yourself (never force if there is pain; instead, learn to work with your body and adapt the movement to your current state). Respect your body and your breath. Also respect those around you: position yourself so you do not bump into anyone while moving;

- Breathing guides the movement: the movement lasts throughout the inhalation and throughout the exhalation.

Poses

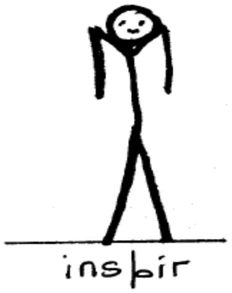 Inhale<br>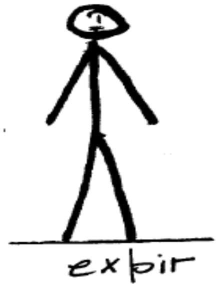 Exhale	**1. Shoulder Contraction/Relaxation**<br>Sit on the edge of a chair, back straight and not leaning, or stand.<br>- Inhale: raise your shoulders toward your ears, then pause for a few seconds to feel the contraction in your shoulders.<br>- Exhale: release your shoulders while exhaling and feel the relaxation.<br>Repeat 3 or 4 times, then roll your shoulders forward and backward several times, mobilizing the upper back.<br>Pause for a moment and observe sensations in your shoulders and upper back. Welcome yawns and sighs if they arise.
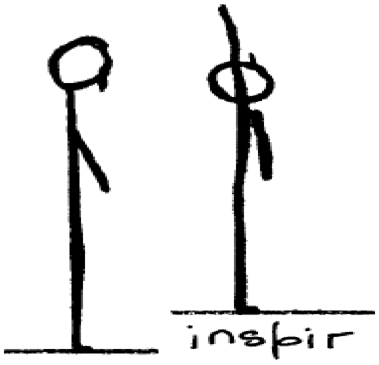 Inhale<br>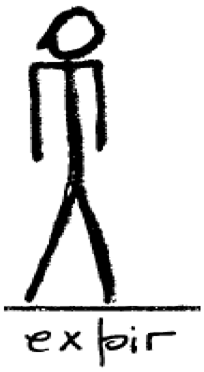 Exhale	**2. Thumb Gaze Exercise**<br>Starting position: standing or sitting on the edge of a chair without leaning back, arms relaxed.<br>Look at your right thumb throughout the exercise:<br>- The right hand rises in front during inhalation until above the head,<br>- Lowers to the side during exhalation,<br>- Rises to the side during the next inhalation,<br>- Then lowers in front during exhalation.<br>Repeat 2 or 3 times on the same side. Then look at your left thumb and repeat the sequence.<br>Afterward, let your arms hang and observe sensations in your neck, shoulders, arms, hands, and fingers while breathing calmly. Notice any emotions. If needed: yawn, sigh, and/or roll your shoulders forward and backward.

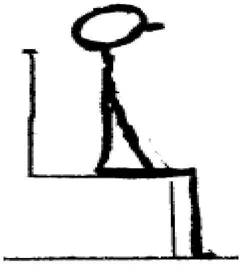<br>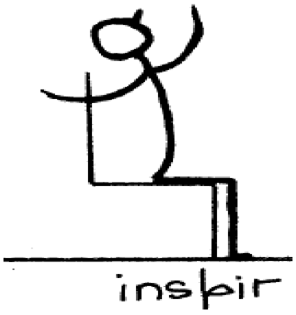<br><br>Inhale<br>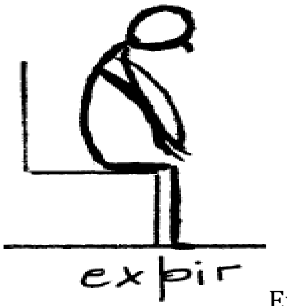<br><br>Exhale<br>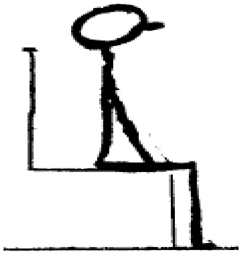	**3. The Flower**<br>Starting position: seated on the front of a chair, not leaning back, feet flat on the floor, knees slightly apart.<br>- Inhale: spread your relaxed arms to the sides, shoulders back, chest and face turned upward.<br>- Exhale: bring your hands together back-to-back between your knees, shoulders forward, chest and face turned downward.<br>Repeat 4 to 6 times, then find an intermediate position—stable and comfortable—with your back straight, shoulders neutral, face forward, gaze straight ahead. Welcome any sensations.<br>Then forward bend: cross your arms on your knees, let your torso bend forward, relaxing your neck, shoulders, and upper back. Stay in this posture for 5 or 6 breaths, as long as it remains comfortable, observing sensations in your back and breathing. Then straighten your torso on an inhalation and notice sensations and emotions.
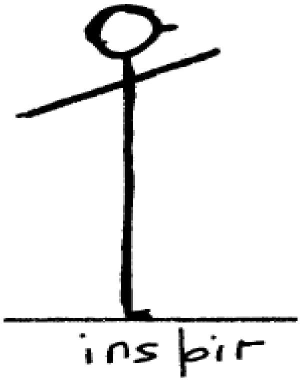<br><br>Inhale	**4. The Mast**<br>Starting position: standing, feet slightly apart, arms in front at shoulder height, palms facing each other.<br>- Inhale: shift your weight toward the front of your feet without lifting your heels, arms open to the sides at shoulder height.<br>- Exhale: shift your weight toward the back of your feet without lifting your toes, arms in front at shoulder height, palms facing each other.<br>During this exercise: look straight ahead and find the limit beyond which heels or toes would lift, causing imbalance. Approach the limit without crossing it.

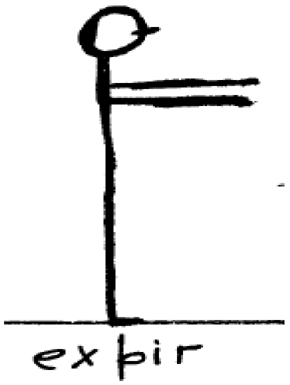<br>Exhale	Then observe sensations and emotions, welcome sighs and yawns.
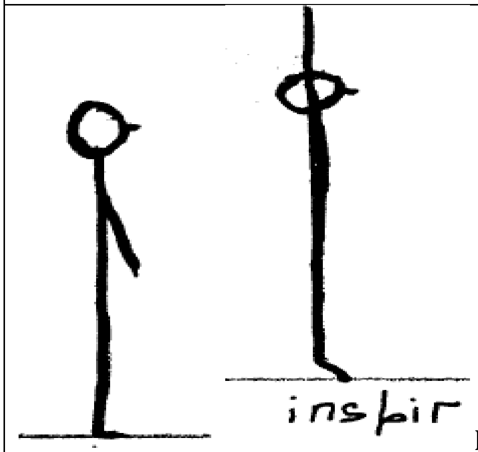<br>Inhale<br>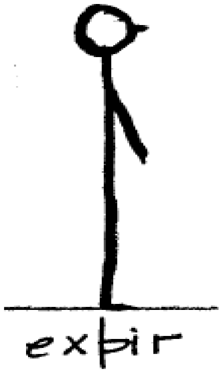<br>Exhale	**5. The Palm Tree**<br>Starting position: standing, feet slightly apart, arms relaxed, gaze straight ahead.<br>- Inhale: raise your arms in front while lifting your heels slightly off the floor (if balance is difficult, keep heels down).<br>- Exhale: lower your arms to the sides as your heels slowly return to the floor (coordinate both movements with your breath).<br>Repeat 4 times, then observe sensations and emotions, welcome sighs and yawns, and roll your shoulders forward and backward.
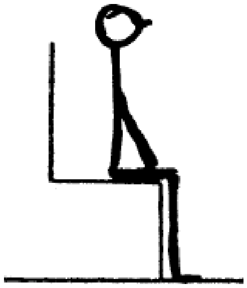	**6. Taraka**<br>Starting position: seated on the edge of a chair, back straight and not leaning, one hand on each thigh, gaze straight ahead.<br>This exercise only moves the eyes; the head stays still.<br>- Look fully to the left, then center,<br>- Fully to the right, then center,<br>- Upper left, then center,<br>- Lower right, then center,

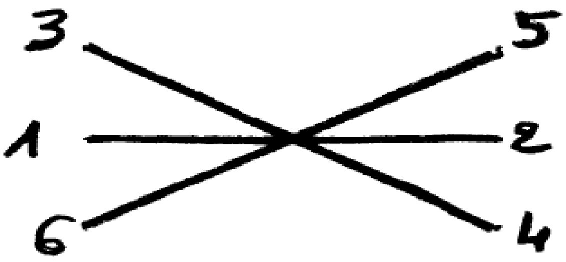	- Upper right, then center,<br>- Lower left, then center.<br>Breathe calmly with your gaze centered, then repeat 2 or 3 times.<br>Then palming: rub your hands together until they feel warm, then cup them gently over your open eyes and observe sensations in your hands, eyes, and around the eyes. Finally, rest your hands on your thighs and take a moment to notice sensations and emotions.
