# Supplementary material for: Case Report: Integrating CBT, hypnosis-based consciousness activation techniques, and yoga-based postural training: a three-pillar approach used for migrant populations
Source: Front Psychiatry. 2026 Jun 26;17:1737072. doi: 10.3389/fpsyt.2026.1737072 (PMC13350056; doi:10.3389/fpsyt.2026.1737072)
Supplement: Supplementary file 4 [file Supplementaryfile4.pdf]

Session number	Time from filling of Beck comumns			The assessment of body flexibility: Arms			
				with diffculty	middle	middle-fexible	flexible

Session number	The assessment of body flexibility: Neck				sleap dairy	
	with diffculty	middle	middle-fexible	flexible	nightime awaking	falling back asleep after nighttime awakenings: time post awaking

Created by: Agnieszka Suchocka Capuano
